# Rapid implementation of a medical student rotation in health systems operations and remote patient care in response to COVID-19

**DOI:** 10.1080/10872981.2022.2067024

**Published:** 2022-05-04

**Authors:** Brian Hilliard, Ronald A Reilkoff, Karyn D. Baum

**Affiliations:** a Medicine and Pediatrics, Division of General Internal Medicine, Department of Medicine, Uniersity of Minnesota Medical School, Minneapolis, Minnesota; b Division of Pulmonary, Allergy, Critical Care and Sleep, Department of Medicine, University of Minnesota Medical School, Minneapolis, Minnesota; c Essentia Health and Adjunct Professor of Medicine, Department of Medicine, University of Minnesota Medical School, Minneapolis, Minnesota

**Keywords:** Triagist, undergraduate medical education, covid-19, health systems, medical student education

## Abstract

Medical schools initially removed students from clinical rotations at the outset of COVID-19 for safety reasons when students were eager to help and health systems needed personnel. In response, we rapidly implemented an innovative 2-week rotation for medical students to participate in health systems operations and care through remote efforts including triage and resource allocation. The curriculum also contained online self-paced educational modules covering topics including ethics, crisis standards of care, and modeling. As the health system needs shifted, so too did learners’ work. One hundred and twenty-five 3rd and 4th-year students completed the experience over 10 months. Learner satisfaction, confidence, and knowledge assessed through pre- and post-rotation surveys showed statistically significant and educationally meaningful improvement. A near uniform change greater than 1 point (on a 5-point scale) was demonstrated upon rotation completion. Blending health systems and educational structures to meet the needs of both creates unique opportunities to educate students in new ways.

After the first case report of coronavirus 2019 (COVID-19) in the USA on 20 January 2020, the disease rapidly spread across the USA^1^. Concern for student safety and personal protective equipment (PPE) availability ultimately led to the removal of students from clinical rotations and on 17 March 2020 the American Association of Medical Colleges and the Liaison Committee on Medical Education formally recommended that students not provide direct patient care^2^.

Effective patient triage is a necessary skill for providers, yet few receive dedicated training in this area^3^. In addition, medical school curriculums rarely provide exposure and offer experiences for students to gain insight into how health-care operations and complex health systems function^4^.

As our system prepared for a surge in patient volumes, we explored opportunities for students to continue their clinical education and engagement whilst contributing in a meaningful and safe way to the pandemic response. To address these disparate needs, we rapidly developed and deployed a novel medical school rotation designed to allow students to provide remote care to support for frontline providers and learn about health system operations and the unique challenges the pandemic presented in a rapidly shifting environment.

A 2-week rotation was designed and coordinated within a pre-existing required sub-internship in Critical Care at a large midwest medical school and its main affiliated health system. The critical care clerkship was chosen as the core objectives aligned closely with the objectives of this novel rotation but with a refined focus on healthcare system management and resource allocation.

The curriculum objectives were administered via both an online and in-person activities and concentrated on interprofessional communication, health systems management, crisis standards of care and medical ethics of rationing care, patient flow concepts, advocacy, and predictive model usage (please see supplemental materials for full course outline, objectives, and student activities). In-person work was performed at our System Operations Center (SOC), which is an innovative, non-clinical center charged with overseeing access, flow, and admissions for patients at seven of the systems’ hospitals. Students were partnered with both clinical and administrative staff, including patient transfer specialists, nursing, and physician and administrative leads. A triage hospitalist, stationed at the SOC as part of the pandemic response, supervised the students and directed their student collaboration and learning. Student duties shifted throughout the 9.5 months according to the needs of the SOC and healthcare system but consistent activities included discharge summary preparation for attending co-signature, intrahospital transfer communication with referring providers with appropriate EHR documentation and screening for rapid allocation of federally distributed EUA therapeutics for COVID-19.

To measure the impact of this rotation and student perceptions, outcomes were assessed through student course evaluations as well as anonymous pre/post surveys centered around self-reported knowledge of the skills focused on during the rotation. All survey questions utilized a 5-point Likert scale with a range from ‘strongly disagree’ to ‘strongly agree.’ Qualitative feedback was obtained for iterative course development and revision. Mann-Whitney U and independent T-tests were used to compare pre and post surveys with similar results. Given the novel approach of this rotation and the rapid implementation, no previous studies were available for comparison to establish a meaningful important difference. The study was approved through the University of Minnesota Institutional Review Board.

One hundred and twenty-five students completed the rotation between 18 March and 31 December 2020, 22 of whom were third year medical students and 103 were fourth year students at the time they completed the rotation. One hundred and four (85%) completed course rotations; 104 completed pre-course surveys and 67 (54%) post-course surveys. Data were reviewed for scoring irregularities (i.e., uniform answers or repetitive patterns). Post-course scoring increased for all categories and questions asked with an observed 22% (1.3 point) [1–6] increase in self-reported knowledge, confidence, and skills across the assessed competencies (see Table 1). In the open-ended portion of course feedback, students consistently reported both educational and personal value in the course due to its nature of unique educational experiences and largely appreciated the opportunity to participate in the pandemic response at a time when many students were removed from on-site rotations.

**Table 1.** Pre- and post-course evaluation scores (all questions on scale of 1–5)

Question	Pre-course (n = 104)	Post-course (n = 67)	*p*-Value
I can effectively determine the appropriate disposition for a patient from the ED or an outside hospital.	2.61	3.63	<0.001
I can efficiently identify key information to determine the safest, most appropriate unit for patients admitted through the ED or transferred from an outside hospital.	2.31	3.56	<0.001
I can effectively weigh the needs of an individual patient with that of the health system to identify the most appropriate hospital within a health system for transfer.	2.11	3.49	<0.001
I am able to effectively communicate across specialties and professions when determining the appropriate disposition for a patient.	2.72	3.63	<0.001
I can identify and navigate the differences in priorities other specialties and institutions may have when determining their rationale for requesting transfer.	2.32	3.51	<0.001
I am comfortable making an initial evaluation of a patient based off chart review and verbal handoff from the transferring provider.	2.79	3.76	<0.001
I am confident in my ability to rely on other interprofessional team members to help determine the appropriate disposition for a patient.	3.21	4.00	<0.001
I understand the various factors determining hospital capacity management during times of crisis.	2.06	3.61	<0.001
I am able to effectively and efficiently communicate the essential information for a transferring patient to an admitting provider.	2.76	3.79	<0.001
Having a physician in the triage role adds value to the health system and patients.	4.14	4.66	<0.001

Limitations of this educational innovation are multifold. First, it was conducted at a single university and its main health system with a dedicated systems operation center which may not be applicable to many organizations or health-care systems. Second, outcomes were self-reported and did not assess any subsequent changes in learner behavior, skills or knowledge, nor did it evaluate patient impact. Third, non-response (46% of post course surveys) may have impacted our results as well as the use of non-validated surveys as the main form of program evaluation. Finally, the surveys were not matched and limited data on triage call volume, remdesivir allocation, and discharge summary preparation was collected.

This paper describes the rapid development of a unique hybrid curriculum for medical students allowing them to safely provide meaningful contributions to COVID-19 pandemic response while also effectively learning about topics often overlooked in medical education. While the specific needs may vary at each institution and over time, training medical students to understand and address health system challenges is essential. Implementing opportunities that serve the dual purpose of educating the next generations of physicians while meeting the needs of our health system during this crisis was effective and well received. Future efforts should include evaluation of patient and system impact, as well as student skills acquisition. This flexible, hybrid model can be utilized by many schools as we adapt to our new world and respond to any subsequent health-care crisis. For future efforts to be successful, early buy-in from both medical school and health system leadership is vital in developing these types of experiences as is identifying learning objectives early and tying them to the student activities.

## Supplementary Material

Supplemental Material
