# Supplementary material for: Rapid implementation of a medical student rotation in health systems operations and remote patient care in response to COVID-19
Source: Med Educ Online. 2022 May 4;27(1):2067024. doi: 10.1080/10872981.2022.2067024 (PMC9090420; doi:10.1080/10872981.2022.2067024)
Supplement: Supplemental Material [file ZMEO_A_2067024_SM8414.docx]

Supplemental Materials:

### Course Description

- Interhospital transfer is a major consideration in all acute care settings and impacts patient and system level outcomes. Additionally, current health care delivery in the acute care setting is marked by limited bed availability and prolonged ED boarding. The intention of this elective is to allow medical students to participate in high level system operations and patient triage from hospitals outside and around the MHealth Fairview system. This includes working with senior system clinical operations leaders and hospital medicine triage physicians to evaluate potential transfers from outside hospitals, coordinate the effective and safe disposition of these patients, and communicate the current status and needs of the patient with an interprofessional team. Students will also aid hospitalists in the care of patients at UMMC by helping prepare drafts of documents such as discharge summaries.
- The MHealth Fairview System Operations Center (SOC) is an innovative center designed to improve efficiency in, access to, and flow through the acute care settings throughout the system. As part of the system’s COVID19 preparation planning, we are accelerating changes already planned and identifying and implementing additional opportunities to assure we are prepared to care for patients should a significant number of patients require care. This is a unique opportunity for a student to be part of fast-paced, system level structure change when it matters most.

### Course Objectives

By the end of the rotation, students will be able to:

- Explain some of the complex factors that determine patient placement within an integrated academic health system comprised of 11 community hospitals and one academic medical center.
- Describe the impacts of variation in patient volumes on health system efficiency and quality
- Efficiently aid physicians from a remote setting in the care of patients through chart work.
- Explain the range of possible impacts that the current COVID-19 pandemic may have upon the health system and identify best practices in the triage and expansion of care delivery during a time of health system crisis.
- Be proficient in the efficient evaluation of a patient’s clinical status and disposition determination in real-time
- Be an effective communicator with an interprofessional team

PCRS competencies

- C0400 - Interpersonal and communication skills
- C0402 - Interprofessional communication
- C0600 - Systems-based practice
- C0602 - Coordinate patient care within a health system
- C0700 - Interprofessional collaboration
- C0204 - Apply principles of epidemiological sciences to populations and patients

### Roles and Responsibilities

- Vet patients for appropriate site placement for Central, East and South Region (Behavioral, Peds, and OB out of scope); looking in chart, using a list of tools provided by the SOC, to direct patients to appropriate sites. Work closely with patient transfer specialists and hospitalists to clarify clinical needs and coordinate in-system reassignments.
- Participation in C4 (statewide patient placement program during COVID surges) through collecting information, contacting potential institutions with availability, and assist in prioritization of patient transfers.
- Review the pending transfer list 4 times per day in conjunction with UMMC flow and reach out to external sites for updates.
- Collaborate with pharmacy students and staff to identify candidates for remdesivir allocations through the state department of health utilizing federal allocation guidelines (this work was preliminary and then verified by staff pharmacists and physicians).
- Review list of patients in EDs for discharge to home, reassignment to other sites using the checklist in conjunction with SOC Triage Hospitalist.
- Learn the role of the patient transfer specialist in case of staff illness.
- As able, work with UMMC hospitalists to identify discharges for the next day and draft (share) discharge summaries.
- Attend project meetings to evaluate the efficacy of the new processes and identify further opportunities.
- Update the reading list for the next student cohort.
- Other duties as assigned and agreed to by the Medical School.

We do not have specific numbers for the above responsibilities, but the workload was significant in correlation with hospitalization rates.

Questions utilized for open ended responses:

- What worked well on this rotation?
- What would you change about this rotation to make it better?
